# Increasing costs from bladder cancer in the Brazilian Health System: the role of establishing public health policies

**DOI:** 10.1590/S1677-5538.IBJU.2020.0658

**Published:** 2021-02-03

**Authors:** Fernando Korkes, Fernando Maluf

**Affiliations:** 1 Faculdade de Medicina do ABC Santo André SP Brasil Disciplina de Urologia, Faculdade de Medicina do ABC, Santo André, SP, Brasil; 2 Hospital Israelita Albert Einstein Departamento de Urologia São Paulo SP Brasil Departamento de Urologia, Hospital Israelita Albert Einstein, São Paulo, SP, Brasil; 3 Hospital da Beneficencia Portuguesa de São Paulo São Paulo Brasil Hospital da Beneficencia Portuguesa de São Paulo, São Paulo, Brasil

## COMMENT

### BC number and treatment advances

Bladder Cancer (BC) is not a very common malignancy. In Brazil, there are 10.640 new cases estimated for 2020, representing 1.6% of all malignancies (1, 2). In Brazil, aging population has been associated with a significant increment in the number of patients with BC during the last decade (3). During this period, an increment of 44% in the number of hospital admissions due to BC have occurred (3). Apart from that, unfortunately, treatment of the initial phases of BC is complex and underperformed in the public health system (SUS) (4). The main reasons are the limited access to the initial evaluation, diagnosis, initiation of treatment, and treatment availability, resulting in a significant number of patients with more advanced disease (3). BCG shortage is another worldwide problem associated with more disease progression to advanced disease.

Fortunately, during the last years, we have witnessed significant advances in new treatments for this disease. Immunotherapies, targeted therapies, diagnostic and staging tests, and several local technologies have been associated with lifespan and quality of life improvements for these patients (5). In the last three years, five immunotherapies (avelumab, atezolizumab durvalumab, pembrolizumab, and nivolumab) and one targeted therapy (erdafitinib) have been approved for the treatment of patients with BC (5). These medications can now be used in a variety of scenarios, from non-muscle invasive to first-line metastatic, maintenance or second-line metastatic treatments. Furthermore, there are also new medications being approved (5, 6). There is no doubt that we can now treat these BC patients better and better.

### Costs with BC treatment

**Figure 1:**
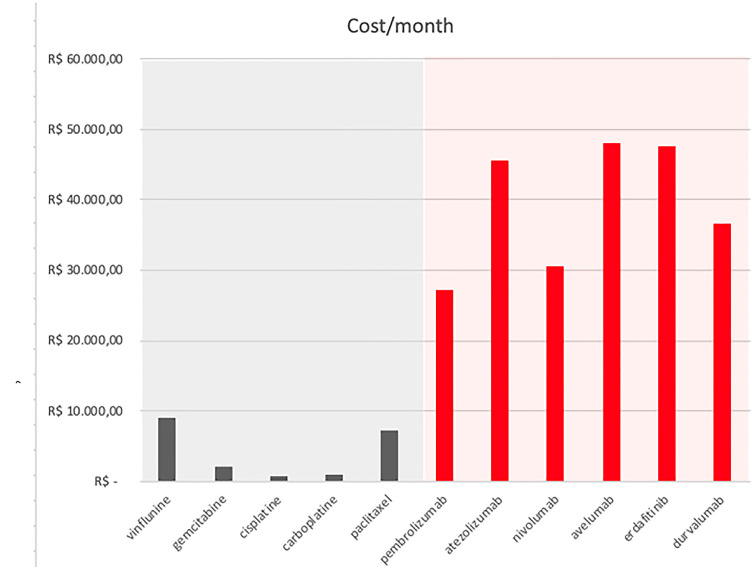
FDA (USA Food and Drug Administration) approved medications to treat BC currently available in Brazil and their estimated costs in R$ in 2020. In gray medications available before 2016. In red, medications available since 2016.

However, these scientific advances come at high costs (Table-1). Despite its relatively low frequency, BC treatment is associated with elevated expenses. It is one of the malignancies with the highest lifetime treatment costs per patient (7). In the USA, it is estimated that US$ 4 billion are spent each year to treat BC (8). And these exceedingly high expenses were calculated when most BC treatments were significantly cheaper than they are today (Figure-1). Not only are the medications associated with a remarkably higher monthly cost, but they are also used for significantly longer periods. This costs to the SUS can exponentially increase, and here are some of the reasons.

**Table 1:** Predicted cost increments on bladder cancer treatment.

Reason to increase costs		Cost increment
Number of BC cases	yearly increment of 23% during the last decade in Brazil; (3)	23%
Cost of treatment/patient	increased 2.367% (from R$ 40.177.00 to R$ 951.071.20) if we follow the recent advances in life expectancies observed in recent studies with immunotherapeutics in metastatic disease (13)	2.367%
Judicialization in SUS	Increased at a 30% / year in the last years (14)	30%
Costs of buying individually	Costs of medication bought individually and not in large scale are four times higher.	400%
**TOTAL increment**	**Potential increment in costs with BC treatment during the next years**	**15.141%**

Font: Site https://consultaremedios.com.br. Estimated values for a 70Kg adult.

### Judicialization in the SUS

Fortunately, in Brazil, health is a citizen's fundamental right guaranteed by the Constitution of 1988. The Universal Health System (Sistema Único de Saúde - SUS) was created to treat every single Brazilian that need healthcare. Despite advances brought by this Constitutional right, many treatments are not promptly incorporated into the routine care in the public system. In such a context, some citizens search for federal courts to obtain new and costly medications. The so-called judicialization of health involves court decisions that require the government to provide health products and services based on the Constitutional right to health. And these lawsuits are considered a significant challenge for the SUS (9).

**Figure 2:**
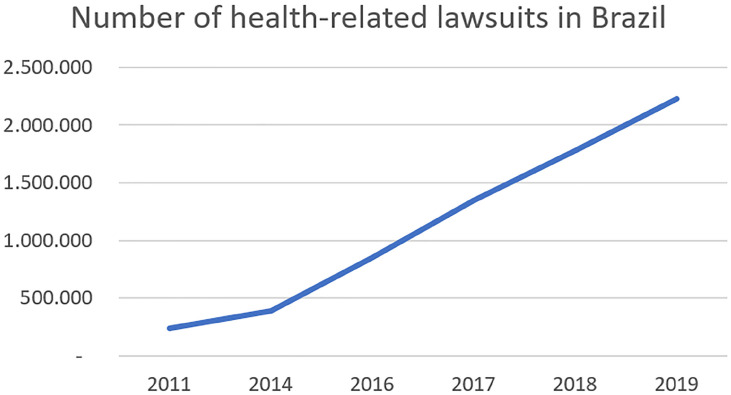
Health-related lawsuits in Brazil from 2011 to 2019 (15).

Even though healthcare judicialization in the SUS has its social and positive role, it also brings significant financial consequences to the Union and to society. In 2019 there were 2.228.531 health-related lawsuits in Brazil. Of those, 980.975 (44%) were motivated by granting access to medications or treatments in the public health system (SUS) (9). During the last eight years, the number of health-related lawsuits has increased astonishing 925% (Figure-2). The public expenditures with health-care judicialization have increased 4.600% from 2007 to 2018 (9). In 2018, the Health Ministry was forced to spent R$ 1.3 billion through judicialization to treat only 1.300 patients. This number represents more than 1% of the SUS annual budget. Among the 20 drugs that represent the higher expenditures for the SUS through judicialization, seven are associated with cancer treatment, and the National Commission incorporated none of them for the Incorporation of Technologies to the SUS (CONITEC) (9).

There is no doubt that requests for new innovative products offered by the pharmaceutical industry pose a significant challenge for the public health system. Nevertheless, there are means that patients can have access to these effective medications. It is fundamental to balance individual rights with society's necessities and, more importantly perform a cost-effective analysis. Moreover, there are several possible solutions to these problems.

### Possible solutions

Centralization programs have been proven to reduce costs and improve outcomes when treating BC (10). Creating reference centers is beneficial not only for the treatment of an individual patient but also for bringing these patients to centers where more strict protocols can be followed (11). New medications can be prescribed when they are beneficial, but judicialization tends to be lower in this setting (9).

Additionally, creating and following strict protocols can help to predict costs. The dynamic inclusion of new drugs to the SUS is essential in the development and approval of new therapies. SUS does not have established protocols for the treatment of most diseases. This flaw favors judicialization. With judicialization, public managers cannot predict the health budgets and might need to relocate provisioned resources. On the other hand, costs for acquiring medications in a large scale are about four times lower than buying individual medications. With provision and scale, negotiations with the pharmaceutical industry could lower even more these costs. Also, there are many mathematical models to lower drug costs, such as payment only when the medication has provided clinical benefit such as the example implemented with spinal muscle atrophy. Also, the government can buy generic drugs when they are available, and in the event they are not, negotiate with the pharmaceutical companies to buy that specific drug according to the lower price rate worldwide.

Clinical Trials are also always an attractive solution for patients with advanced cancer. Fomenting and international trials to come to Brazil can lower the burden of BC's treatment. They help to reduce costs to the system and contribute to the progress of science. In this regard, cooperative groups such as the Latin American Oncology Cooperative Group has been launched many protocols in solid tumors such as bladder cancer (12).

Even though BC is relatively uncommon, it is expensive. Furthermore, there is a paucity of public health policies aiming at these patients. The Union and the medical society must sum efforts to assess this situation. Otherwise, the combination of increments in BC cases, treatment costs, and judicialization can lead to a collapse in the SUS shortly.
